# A Comparison of Outcomes for Adults and Children Undergoing Resection for Inflammatory Bowel Disease: Is There a Difference?

**DOI:** 10.1155/2014/410753

**Published:** 2014-03-27

**Authors:** Christine M. Mcmullin, Jonathan Morton, Saranya Vickramarajah, Ewen Cameron, Miles Parkes, Franco Torrente, Robert Heuschkel, Nicholas Carroll, R. Justin Davies

**Affiliations:** ^1^Cambridge Colorectal Unit, Addenbrooke's Hospital, Cambridge University Hospitals NHS Foundation Trust, Cambridge Biomedical Campus, Hills Road, Cambridge CB2 0QQ, UK; ^2^Department of Gastroenterology, Addenbrooke's Hospital, Cambridge University Hospitals NHS Foundation Trust, Cambridge Biomedical Campus, Hills Road, Cambridge CB2 0QQ, UK; ^3^Department of Paediatric Gastroenterology, Addenbrooke's Hospital, Cambridge University Hospitals NHS Foundation Trust, Cambridge Biomedical Campus, Hills Road, Cambridge CB2 0QQ, UK; ^4^Department of Radiology, Addenbrooke's Hospital, Cambridge University Hospitals NHS Foundation Trust, Cambridge Biomedical Campus, Hills Road, Cambridge CB2 0QQ, UK

## Abstract

*Background*. The incidence of inflammatory bowel disease (IBD) is increasing in the paediatric population. Since 2007, a single surgeon whose main practice is in the treatment of adults has performed surgery for IBD in adults and children within two dedicated multidisciplinary teams. Our aim was to assess and compare outcomes for adults and children following surgery for IBD. *Methods*. Analysis of a prospectively collected database was carried out to include all patients who had undergone resectional surgery for IBD between 2007 and 2012. *Results*. 48 adults and 30 children were included in the study. Median age for children was 14 years (range 8–16) and for adults was 33.5 years (range 17–64). Median BMI was 23 (range 18–38) and 19 (range 13–29.5) in adults and children, respectively (*P* < 0.001). Laparoscopic resection was performed in 27 (90%) children and 36 (75%) adults. Postoperative complication rates were comparable, 11 (23%) in adults versus 6 (20%) in children (*P* = 1.00). *Conclusion*. Resectional surgery for IBD in children has outcomes that compare favourably with the adult population, with the majority of cases being performed by a laparoscopic approach.

## 1. Introduction

The incidence of inflammatory bowel disease (IBD) is rising in Western Europe and North America [1]. Since the 1980s, there has been an increase in the proportion of patients presenting with symptoms of IBD (3.6% for ulcerative colitis and 10.3% in Crohn's Disease) [1]. Inflammatory bowel disease tends to predominantly affect young people, with a peak incidence between the ages of 15 and 35 years [2]. The incidence of IBD in children in the United Kingdom under 16 years is 5.2 per 100,000 individuals per year [2, 3].

Despite recent advances in medical therapy for both ulcerative colitis (UC) and Crohn's disease (CD), there is still a need for surgery in patients who develop appropriate symptomatology or develop complications of their disease or its treatment. Data suggest that 30–40% of patients with UC and 70–80% of patients with CD will need surgery during their lifetime [4]. As the general population continues to increase and specialist services such as adolescent IBD services are centralized, adult surgeons are increasingly presented with younger patients being considered for surgical intervention.

The aim of this study was to evaluate and compare the outcomes of children and adults undergoing resectional surgery for IBD in a tertiary referral centre.

## 2. Methods

Since 2007, a single adult surgeon has been performing resections for inflammatory bowel disease (IBD) in children and adults within separate, dedicated adult, and paediatric multidisciplinary IBD teams. Outcomes for all patients undergoing surgery between December 2007 and July 2012 were prospectively recorded on a dedicated, password-protected, electronic database. All patients who had undergone resectional surgery for IBD as well as ileal pouch-anal anastomosis were included in the study. Those patients having perianal procedures, stoma formation, or strictureplasties alone were not included. Children were defined as those aged 16 years or less. The outcome measures assessed were complications, length of stay, median follow-up, and median time to disease recurrence.

## 3. Statistics

Statistical analysis was performed using InStat 3.0 (GraphPad Software Inc., CA, USA). The Mann-Whitney test was performed for nonparametric data and Fisher's Exact test for two-by-two contingency tables.

## 4. Results 

A total of 78 patients (30 children and 48 adults) underwent surgery (see Table 1). The median age for adults and children was 33.5 years (range 17–64 years) and 14 years (range 8–16 years), respectively. The difference in median BMI between the groups was statistically significant (*P* < 0.001), with adults having a higher median BMI (23) than children (19). Twenty-six (54%) adults and 20 (67%) children had CD. Of these, 6 were smokers, 5 of whom were in the adults group.

Table 2 demonstrates the operative differences between the two groups. Twenty-seven (90%) children and 36 (75%) adults underwent laparoscopic resection (*P* = 0.14). Six (16.7%) operations were converted to open in the adult group compared to none in children (*P* = 0.03). The median operative time in the adult laparoscopic group was 210 (range 80–300) minutes compared to 165 (range 40–300) minutes in children. The median operative time for open and laparoscopic surgery in adults was 210 (range 60–420) minutes compared to 175 (range 40–300) minutes in children (*P* = 0.03). Ten operations were performed as emergencies, six of which were in the adult group. Twenty-three patients in the adult group underwent subtotal colectomy and ileostomy and, of these, 13 (56.5%) went on to have ileal pouch-anal anastomosis (IPAA) compared to 4 (26.7%) of 15 subtotal colectomy and ileostomy in the children's group to date. Median length of stay was 5 days in adults versus 6.5 days in children (*P* = 0.31) with the main cause of delay in children being stoma education.

Postoperative complication rates were comparable (see Table 3), 11 (23%) in adults' versus 6 (20%) in children (*P* = 1.00). There were two anastomotic leaks, both occurring in the adult population.

Median follow-up in adults was 19 (range 1–55) months and in children 21 (range 1–52) months. In terms of disease recurrence in CD, there were 6 patients with neoterminal ileal recurrence in the adult group with a median time to recurrence of 10 months (range 4–31 months) compared to one disease recurrence in the children with time to recurrence of 14 months. Twenty-seven (56%) adult patients were on immunosuppressants or steroids prior to surgery. Of these, 4 developed postoperative complications (none of which were infective). Twenty-four (80%) paediatric patients were on immunosuppressants or steroids before surgery. Of these, 5 developed postoperative complications (1 of which was a wound infection).

## 5. Discussion

The surgical management for children with IBD, particularly adolescents, tends to fall in the transition from paediatric to adult colorectal surgical services. The UK guidelines on the management of IBD in paediatric patients recommend close collaboration between gastroenterologists and a surgeon with experience in paediatric IBD [3] in order to be able to offer the patient the best and safest surgical option available. Although IBD in paediatric patients may present the same way as adults in terms of symptoms, the emotional, mental, and developmental needs of this population are different to that of an adult cohort. Inflammatory bowel disease can cause growth failure in children, and side effects from the medical treatment available can contribute to growth retardation and also bone demineralisation [3]. It is therefore important for paediatric and adolescent patients with IBD to be managed in a specialist multidisciplinary team in order to optimize the patients' medications and aid in decision making as well as transitioning to adult services [3]. Our data suggest that children with IBD can be successfully managed by an experienced adult colorectal surgeon within a paediatric MDT setting. This is demonstrated by the lack of difference in outcomes between the child and adult groups. There are advantages to an adult surgeon performing these resections from a continuity of care perspective, (patients often undergo staged procedures) [5]. For example, patients with UC who undergo subtotal colectomy for fulminant colitis will be known to the same team who can help guide patients and their guardians through the often difficult decision making process of ileoanal pouch surgery in this age group. Surgeons who regularly operate on adult patients with IBD are also well versed in the often multiple different potential operative strategies available, as well as being experienced in the occasional unexpected operative findings that can occur in patients undergoing surgery for CD despite best attempts at preoperative investigation.

Laparoscopic surgical resection for IBD in adults is a well-established practice, with short-term outcomes superior to those of open surgery [5–11]. The main advantages offered by laparoscopic surgery over open surgery include reduced intraoperative blood loss, better cosmetic outcome, reduced postoperative pain, and shorter hospital stay [6–9, 11]. In a previous study at our institution, we compared outcomes of laparoscopic and open surgery for IBD resections in children and found that there was no statistical difference between the two approaches in children [5]. Fischera [11] report a conversion rate of 10.9% (*n* = 125) in a study of adult patients undergoing laparoscopic resection for CD. Mattioli and colleagues [6] report a 0% (*n* = 16) conversion rate in children undergoing laparoscopic surgery for IBD. Our study highlights the point that median operative times are lower in children, likely a reflection of the higher median BMI found in the adult group. A laparoscopic approach in paediatric patients with IBD does therefore appear safe and has outcomes that compare well to the adult population. In addition, an experienced adult colorectal surgeon can perform single incision laparoscopic surgery in paediatric patients with ulcerative colitis and we have previously highlighted this in a case of a 13-year-old girl with UC [12].

Postoperative complications in patients with IBD are relatively common and the reported complication rates vary. The main postoperative complications are anastomotic leak [5], small bowel obstruction [13, 14], wound infection [5, 10, 13, 14], and venous thromboembolism (VTE) [10, 14]. The long term complications are incisional and parastomal hernias [8, 10] as well as adhesional small bowel obstruction [8]. Schaufler et al. [14] report an 8% wound infection rate in their study and Patton et al. [13] report a rate of 13%. In addition, Patton and colleagues report a parastomal hernia and anastomotic leak rate of 3% for each condition [13].

Patients with IBD on steroid therapy are particularly at risk for the development of VTE [14]. Wallaert et al., found that 224 of 10,431 patients with IBD developed 242 VTEs within 30 days of surgery and on average the diagnosis was made at day 10 [15]. Soon et al. report one incidence of VTE in a retrospective study of 30 paediatric IBD patients [16]. We had 3 VTE events, all of which occurred in the adult group: a pulmonary embolus, a sagittal sinus thrombosis, and a portal vein thrombosis. All of the patients in both the adult and paediatric groups received appropriate thromboprophylaxis following surgery.

In our study, two paediatric patients went back to theatre for small bowel obstruction. Patton et al., in a retrospective study assessing the postoperative outcomes for paediatric patients undergoing a colectomy with UC, found that 8 (26%) out of 31 patients developed small bowel obstruction [13]. Schaufler et al. in their series of 51 patients after colectomy for colitis reported that 10 (19%) patients developed small bowel obstruction and 3 required surgical intervention [14]. The percentage of IBD patients who go on to develop small bowel obstruction postoperatively ranges from 3% to 19% in three different studies [10, 13, 14]. Although these studies identified small bowel obstruction as an early complication, they did not clearly define what diagnostic criteria were used to make the diagnosis. The inclusion of postoperative ileus in the group of small bowel obstruction may account for the relatively high rates seen in the studies above [13, 14].

Despite 56% of adults and 80% of paediatric patients in our study being on immunomodulators at the time of surgery, infective complication rates in both groups were low. Subramanian et al., in a systematic review of the postoperative outcomes of IBD patients treated with immunomodulators, found that their use was not associated with an increased risk of infectious postoperative complications after surgery for IBD [17]. Further studies in the paediatric population assessing the impact of immunosuppression on infective complications have found no increase in postoperative infectious complications [13, 14].

The main limitation of our study is that it is a single surgeon series.

## 6. Conclusion

Postoperative outcomes for resectional IBD surgery in paediatric patients are comparable to those seen in adults. In addition, our experience suggests that a colorectal surgeon, whose main practice is in the adult population, can provide a safe service for paediatric patients as a member of the paediatric and adult IBD multidisciplinary teams. This approach also facilitates an effective transition from paediatric to adult services, allowing good ongoing continuity of care.

## Figures and Tables

**Table 1:** Patient demographics (median values).

	Adults *n* = 48	Children *n* = 30	*P* value
Age (years)	33.5 (17–64)	14 (8–16)	
BMI	23 (18–38)	19 (13–29)	*P* < 0.001
ASA	2 (2-3)	2 (2-3)	
Operation time (minutes)	210 (60–420)	175 (40–300)	*P* = 0.03
Patients on preoperative immunomodulators	27 (56%)	24 (80%)	
Length of stay in days	5 (2–80)	6.5 (3–16)	*P* = 0.31
Median follow-up in months	19 (1–55)	21 (1–52)	

**Table 2:** Operation details.

	Adults *n* = 48	Children *n* = 30	*P* value
Operation time (minutes)	210	175	*P* = 0.03
Laparoscopic procedures			
Number of patients	36 (75%)	27 (90%)	*P* = 0.14
Ileocaecal resections	11	10	
Right hemicolectomy	1	4	
Subtotal colectomy	16	10	
Single port subtotal colectomy	4	3	
Ileal pouch-anal anastomosis (IPAA)	6	2	
Small bowel resection	2	0	
Converted to open	6	0	*P* = 0.03
Open surgery			
Subtotal colectomy	3	3	
Ileocaecal resections	4	0	
Right hemicolectomy	4	0	
Ileal pouch-anal anastomosis (IPAA)	7	2	
Small bowel resection	1	0	

**Table 3:** 

	Adults *n* = 48	Children *n* = 30	*P* value
Complications			
Total	11 (23%)	6 (20%)	*P* = 1.00
Anastomotic leak	2	0	
Venous thromboembolism	3	0	
Perforated Duodenal Ulcer	2	0	
Wound infection	2	2	
Ileus	1	0	
Tuboovarian abscess	1	0	
Small bowel obstruction	0	2	
UTI	0	2	
Disease recurrence			
Total	7	1	
Neoterminal ileum	6	0	
Median time to recurrence (months)	10 (4–31)	14 (N/A)	
